# Twinning–Detwinning in Pre-Compressed and Thermally Treated ZX10 and ZN10 Alloys

**DOI:** 10.3390/ma13245605

**Published:** 2020-12-08

**Authors:** Daria Drozdenko, Patrik Dobroň, Klaudia Fekete, Sangbong Yi, Jan Bohlen

**Affiliations:** 1 Department of Physics of Materials, Faculty of Mathematics and Physics, Charles University, Ke Karlovu 5, 12116 Prague, Czech Republic; dobronp@karlov.mff.cuni.cz (P.D.); fekete@karlov.mff.cuni.cz (K.F.); 2 Nuclear Physics Institute, The Czech Academy of Sciences, Hlavní 130, 250 68 Řež, Czech Republic; 3 MagIC-Magnesium Innovation Centre, Helmholtz-Zentrum Geesthacht, Max-Planck Str. 1, D21502 Geesthacht, Germany; sangbong.yi@hzg.de (S.Y.); jan.bohlen@hzg.de (J.B.)

**Keywords:** magnesium alloys, twin boundaries, precipitation, twinning, detwinning

## Abstract

The deformation behavior of extruded Mg alloys with a Ca or Nd addition (up to 0.5 wt.%) is addressed with respect to a specified thermo-mechanical treatment, realized by pre-compression and subsequent heat treatment at intermediate temperature. The twinning–detwinning process is discussed with respect to the initial texture and applied heat treatment. Isothermal aging leads to precipitation and segregation along twin boundaries and dislocations in the pre-compressed Mg alloys, and, thus, variation in the mobility of twin boundaries (TB) is observed in the investigated alloys. Despite individual scenarios of TB mobility in particular grains, in general, the same TB mobility modes are observed in the alloys independently on Ca or Nd alloying. The microstructure development, particularly the twin volume fraction and the mobility of tensile {10-12} twin boundaries, is tracked using scanning electron microscopy, including backscattered electron (BSE) imaging and electron backscatter diffraction (EBSD) mapping.

## 1. Introduction

Mechanical twinning plays a key role in the deformation behavior of Mg alloys. The reorientation of the original lattice and introducing twin boundaries (TB) give rise for the activation of other deformation mechanisms (dislocation glide, detwinning, re-twinning) [1,2]. For instance, dislocation slip restrained in its activity in the originally oriented lattice can become operative in the re-oriented one. At the same time, TB introduce nuclei for <c+a> dislocations [2], which contribute to deformation along the <c> axis necessary for plastic deformation and usually require high applied stresses. Meanwhile, twin lamellae subdivide grains, act as non-dislocation barriers and contribute to the hardening of the material. The polar nature of twinning together with a strong texture of extruded Mg alloys, formed during processing, results in a significant asymmetry of mechanical properties [3,4,5,6,7]. Thus, the yield strength (YS) asymmetry with a lower compressive YS compared to the tensile YS (i.e., CYS < TYS) is a characteristic feature of extruded Mg alloys [8]. Application of pre-straining along a certain direction can introduce a new texture component formed by twins, which would lead to a modified texture and, therefore, to reduced or even reversed YS asymmetry (CYS > TYS) [7,9,10,11,12,13,14,15]. Moreover, thermo-mechanical treatment (i.e., with concurrent precipitation hardening) realized by pre-strain followed by heat treatment has become recently popular in order to improve the strength of Mg alloys by texture modification and grain refinement [16,17].

The most common twinning system in Mg alloys is the {10-12} <10-11> extension twinning with a reorientation of the original lattice by 86.3°. This twin system is activated either during tensile loading along the *c*-axis of the hcp unit cell or during compressive loading perpendicular to the *c*-axis. Thus, in extruded Mg alloys having basal planes preferentially oriented parallel to the extrusion direction (ED), the {10-12} <10-11> extension twinning is activated during compression along ED. With further loading in the same strain path, the twins will grow in length and thickness. During the subsequent unloading and reverse loading, the existing twins shrink and eventually disappear, the so-called detwinning process [2,3,18,19]. Detwinning can be realized either by the migration of existing TB to the interior of the twin (twin shrinkage) or by secondary twinning inside the existing twin. The secondary twins can have the same orientation as the original matrix, i.e., during pre-compression and further reverse loading, an identical twin variant is activated. The first way of detwinning (twin shrinkage) has been widely determined in Mg alloys, using ex-situ and in-situ methods, during cyclic loading [3,20,21,22,23,24,25,26] or changing strain path [7,19,27,28]. Besides, the theoretical calculation of the twinning–detwinning mechanism using visco-plastic self-consistent (VPSC) modeling has been presented, e.g., in [29,30,31]. It has been reported that the mobility of TB with its complexity depends on several factors, such as the distribution of the dislocation density [32,33], grain size [20,34], twin size [35], solute atoms [11,36], precipitates [37,38,39,40], and deformation condition [7,23,25,27].

The extension twinning is accompanied by the development of significant residual back stresses and friction stresses as a result of load redistribution between soft and hard grain orientations for further slip [12,41]. To reduce the residual tensile stresses inside the twins without affecting the twin structure, annealing at 200 °C for 30–120 min was applied on rolled AZ31 [41]. The annealing allowed to prolong the activity of basal slip and increased the activation stress for detwinning, which led to improving the YS and elongation. However, it was reported [41,42] that even after prolonged annealing the twin growth and shrinkage are still facilitated. At the same time, heat treatment can lead to precipitation hardening, as reported for Mg–5Zn [40] and Mg–Zn–Ca alloys [43]. Solute segregation and precipitation can profoundly affect the propagation and nucleation of deformation twins quantitatively and qualitatively (the amount and size) [44,45]. Introducing solute segregation and precipitation at TB also modifies the contribution of twinning to plastic deformation. Particularly, the periodic segregation of solute atoms of Zn and Gd in fully coherent twin boundaries has been reported to provide a pinning effect for TB leading to the strengthening of the material [46]. Similarly, the segregation of Al and Zn atoms along TB has been reported to suppress the detwinning in AZ31 alloy [47]. Further, the strengthening of thermo-mechanically treated Mg–0.3Zn–0.1Ca sheets has been ascribed to the pinning of gliding dislocations at Guinier–Preston (GP) zones and possible solute atom segregation to the dislocations [48]. In this context, Cui et al. investigated the effect of short time annealing on the deformation behavior [13] and the damping capacity of Mg alloys [14,33]. Therefore, heat treatment significantly affects the mobility of TB, and by the appropriate selection of the heat treatment parameters (e.g., annealing time) TB can be stabilized or mobilized [13]. A recent study on binary Mg–1 wt.%Zn alloy reports that after isothermal aging at 200 °C for 8 h, some TB continue to move, while some become immobile, and detwinning (during reverse loading) is realized by nucleation of new twins inside the existing one [49]. Despite the effort to explain the effect of heat treatment (annealing or aging) on twin growth and shrinkage, i.e., the TB mobility, it is still not completely clear how intermediate heat treatment can affect the yielding behavior during the subsequent mechanical loading.

The aim of the present study is to determine the mechanical behavior of extruded Mg–Zn alloys with an addition of Ca and Nd with respect to the specified thermo-mechanical treatment realized by pre-compression and followed by isothermal aging. Alloying elements were selected in order to achieve relatively weak textures of the investigated alloys. Further, the strengthening ability stimulated by thermo-mechanically treatment in ZN10 with its more economical alternative represented by ZX10 can be directly compared. The novelty of the work lies in revealing a potential TB pinning effect caused by intermediate heat treatment on the ongoing twinning–detwinning mechanism in either a continuous compression or reverse tensile loading of extruded Mg–Zn–Ca(Nd) alloys with variation in the texture. The detailed analysis of TB is provided by backscattered electron (BSE) imaging and electron backscatter diffraction (EBSD) mapping on the same observation area as far as available. Even though the precipitation at the twin boundaries and within the matrix has been observed as a result of various heat treatments, the attention is focused on the effect of specified intermediate isothermal aging of the pre-compressed alloys on the active deformation mechanisms (twinning–detwinning mechanism, dislocation slip). The detailed analysis of the precipitates and their interaction with the boundary motion is beyond the main scope of the present study. The obtained knowledge can be used for the design of wrought Mg alloys with enhanced mechanical properties.

## 2. Materials and Methods

**Figure 1:**
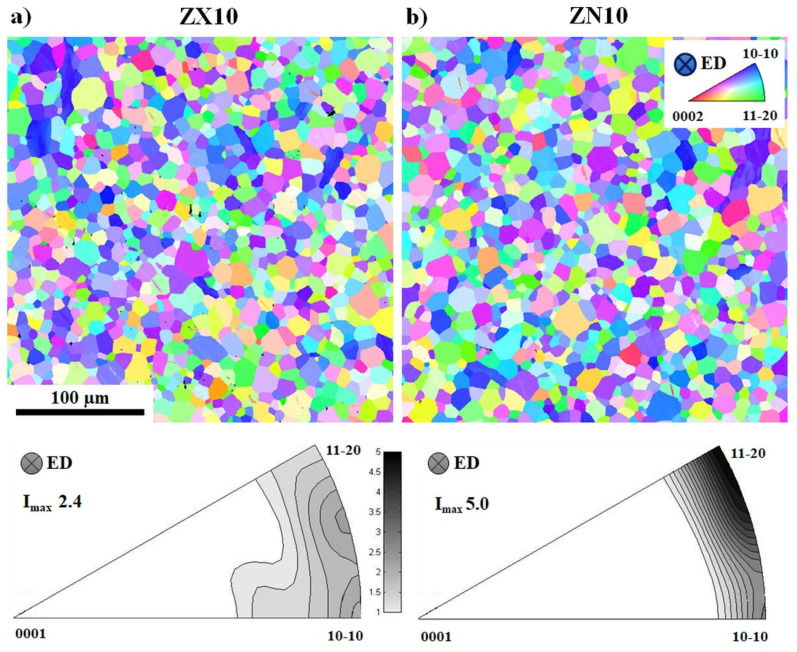
Microstructure and texture of (**a**) ZX10 and (**b**) ZN10 alloys. ED is perpendicular to the image plane of orientation maps and texture triangles.

For the present study, ZX10 (Mg + 1 wt.%Zn + 0.25 wt.%Ca) and ZN10 (Mg + 1 wt.%Zn + 0.5 wt.%Nd) alloys were indirectly extruded to a round bar with a diameter of 10 mm, which refers to an extrusion ratio of 1:25. Extrusion parameters (400 °C and 0.5 mm/s for ZX10; 480 °C and 2.3 mm/s for ZN10) were chosen in order to achieve comparable homogeneous microstructure in the as-extruded condition. An average grain size of 12 ± 2 µm and 13 ± 2 µm in ZX10 and ZN10 alloys, respectively, was revealed, Figure 1. Both alloys have a texture with an alignment of basal planes preferentially parallel to ED, Figure 1. The alignment of basal planes appears to be stronger in ZN10 than in the ZX10 alloy (I_max_ is 5.0 vs. 2.4 m.r.d.). For both alloys, the texture intensities are distributed along the arc between the <10-10>-and <11-20>-poles with a slightly higher intensity at <10-10> pole for ZX10 alloy and at <11-20> pole for the ZN10 alloy.

In order to study the twinning–detwinning mechanism, with respect to the applied intermediate isothermal aging, the following procedure has been performed. The samples were taken in the as-extruded initial condition and pre-compressed up to 3% along the ED (referring to 140 MPa for ZX10 and 120 MPa for ZN10). Then samples received an isothermal aging at 150 °C of 16 h in the case of ZX10 and 48 h in the case of ZN10. This thermo-mechanically treated (TMT) condition is used as the onset for studying the ongoing behavior either in following compression (labeled as “re-compression“) or tension (labeled as “reverse tension“).

The deformation tests were performed using a universal testing machine Instron 5882 (Norwood, MA, USA) at room temperature and a constant crosshead speed giving an initial strain rate of 10^−3^ s^−1^. The sizes of the samples have been specified with respect to the direction of the following loading of the TMT samples: re-compression or reverse tensile loading. In the case of pre-compression and following re-compression, cylindrical samples with a gauge length of 14 mm and a diameter of 9.5 mm were machined along ED. During deformation, the vacuum grease Apiezon M (Manchester, UK) has been used to reduce friction between compression plates and the sample. For pre-compression and the following reverse tensile loading, samples with a gauge length of 13 mm, a diameter of 5 mm, and screw heads on both ends machined along ED were prepared.

For the analysis of the twinning–detwinning process, including the TB mobility, it is necessary to achieve a partially twinned microstructure. Thus, the same level of pre-compression along ED as the one used in previous work on a binary Z1 alloy [49] has been chosen for the investigated alloys. The isothermal aging conditions have been specified with respect to preliminary microhardness investigations (not presented here) aiming to achieve maximum strengthening in each alloy.

The evolution of the microstructure was characterized by a scanning electron microscope (SEM) Zeiss CrossBeam Auriga (Jena, Germany), particularly using backscattered diffraction techniques (BSE imaging and EBSD mapping). EBSD mapping was carried out with a step size of 0.4 µm. Specimens for microstructural observations were polished by standard method up to the usage of diamond paste with the particle size of 0.25 μm and subsequently electropolished in the AC-2 electrolyte (Struers GmbH, Willich, Germany) at 18 V, −40 °C for 60 s.

## 3. Results

**Figure 2:**
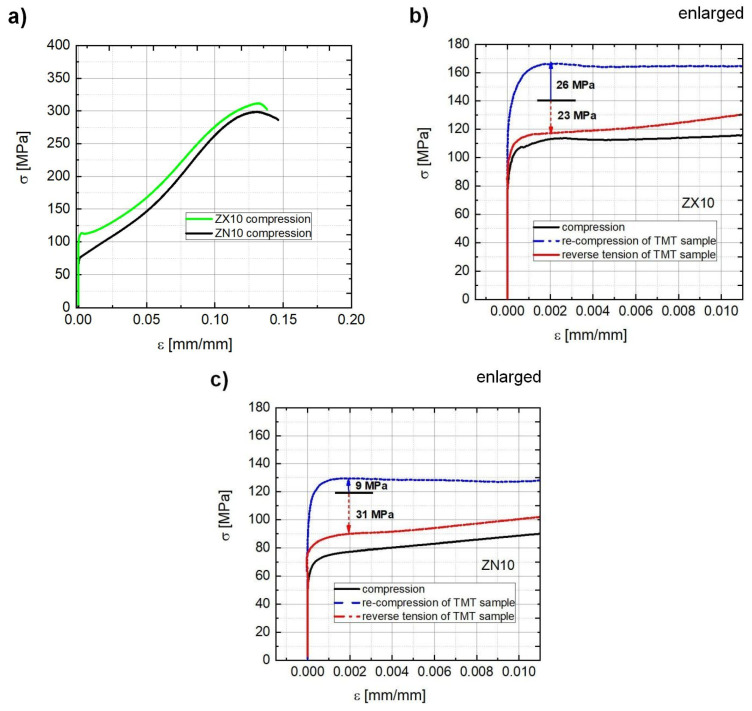
Engineering stress vs. plastic strain dependence for alloys in an as-extruded state (**a**) and in the thermo-mechanically treated (TMT) state: (**b**) ZX10-pre-compression up to 140 MPa (3% of strain) and isothermal aging at 150 °C for 16 h; (**c**) ZN10-pre-compression up to 120 MPa (3% of strain) and isothermal aging at 150 °C for 48 h. Please note that subfigures (**b**,**c**) represent the enlarged region of yielding. Black line in (**b**,**c**) refers to the pre-compression level for each alloy.

The engineering stress vs. plastic strain dependence for the ZX10 and ZN10 alloys in the as-extruded and TMT state is presented in Figure 2. Additionally, the achieved YS (σ_0.2_) values are collected in Table 1. In the as-extruded and TMT states, the ZX10 alloy is characterized by higher onset of plastic flow compared to the ZN10 alloy. The beginning of plastic deformation, up to approximately 3% of strain, is characterized by stronger hardening (steeper hardening slope) in the case of ZN10 in comparison to rather smooth development for ZX10. With further loading above 3% of strain, the hardening behavior (hardening slope and its change with applied stress) is comparable for both alloys. For samples in the TMT state, S-shaped curves are observed for both re-compression and reverse tensile loading, which indicates the twinning activity: twin growth and shrinkage, respectively. During reverse tensile loading of the TMT samples, yielding occurs at lower stresses compared to those during re-compression and, thus, a reversed tension-compression YS asymmetry is observed. Moreover, the increase of CYS is larger with respect to the pre-strain level in ZX10 compared to that in ZN10 (increment of 26 vs. 9 MPa, marked by blue arrows from the pre-strain level in Figure 2b,c). At the same time, a slightly larger drop in the TYS values is observed in ZN10 compared to that in ZX10 (31 vs. 23 MPa, marked by red arrows from the pre-strain level in Figure 2b,c). Thus, besides having the higher CYS in the as-extruded state (113 ± 1 MPa), the ZX10 alloy exhibits a more pronounced hardening effect stimulated by the performed TMT: a larger increase in CYS and a smaller drop in TYS values than those for the ZN10 alloy.

**Figure 3:**
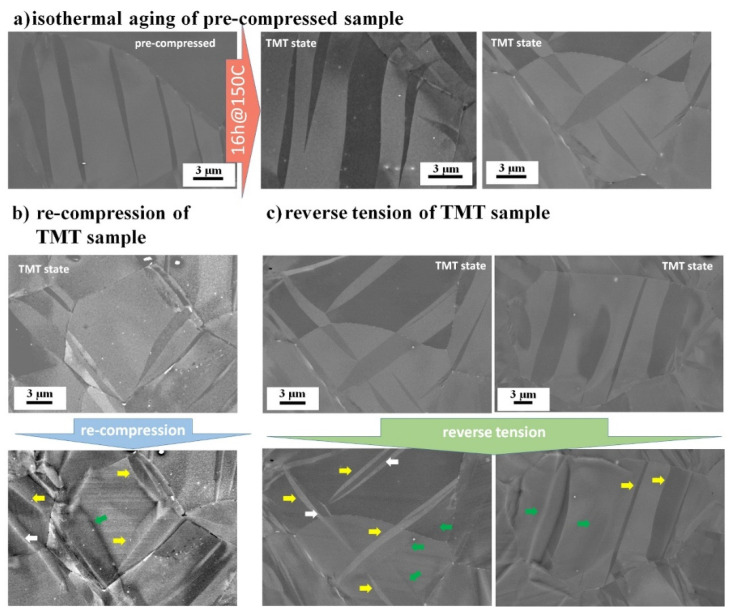
Backscattered electron (BSE) images of the ZX10 alloy: (**a**) with respect to the isothermal aging to achieve TMT state (pre-compression up to 140 MPa and subsequent isothermal aging at 150 °C for 16 h); followed by (**b**) re-compression up to 3% of strain or (**c**) reverse tension up to 2% of strain. ED is horizontal. Colored arrows highlight: yellow-immobile twin boundaries (TB); green–mobile TB; white–newly nucleated twins, accordingly.

**Figure 4:**
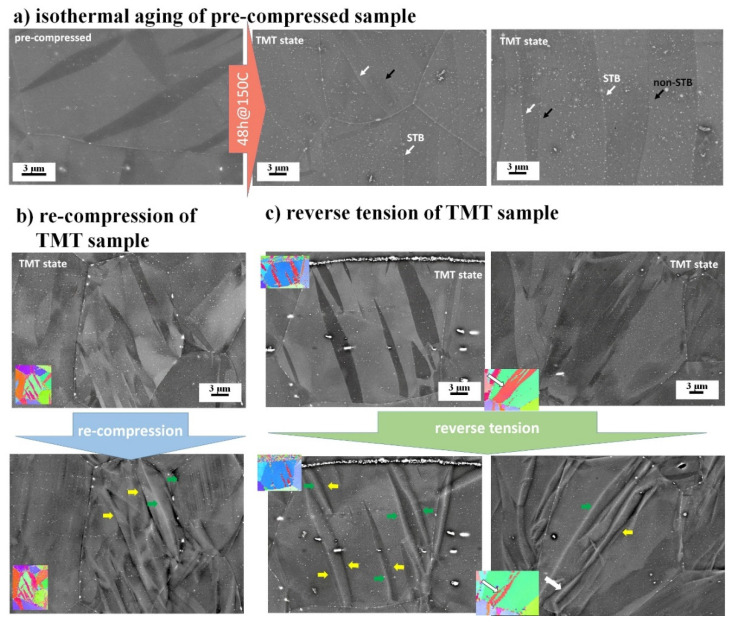
BSE images of the ZN10 alloy: (**a**) with respect to the isothermal aging to achieve TMT state (pre-compression up to 120 MPa and subsequent isothermal aging at 150 °C for 48 h); followed by (**b**) re-compression up to 3% of strain or (**c**) reverse tension up to 2% of strain. STB–segregated twin boundaries. ED is horizontal. Colored arrows highlight: yellow–immobile TB; green–mobile TB; white-newly nucleated twins, accordingly.

An analysis of the microstructure is provided by means of SEM. Particularly, BSE images are used for detailed analysis of segregation and precipitation as a result of intermediate isothermal aging. Further, by comparing BSE images taken from the same area before and after loading, the modes of the TB mobility can be revealed. The BSE images for the ZX10 and ZN10 alloys in different conditions are presented in Figure 3 and Figure 4, respectively. It is seen that the applied isothermal aging leads to a more significant precipitation effect in ZN10 compared to the ZX10 alloy. As the obtained results on precipitation are supported by previous studies on Mg–Zn alloys with Ca and Nd, the composition of such precipitates is not further revealed in this work. However, it appears worthwhile to summarize that in the case of ZX10 alloy, fine coherent precipitates under SEM resolution were observed in our previous work [15]. Further, the detailed transmission electron microscope (TEM) analysis indicated the distribution of precipitates in the grain interior and along the grain and twin boundaries with respect to a different heat treatment. The large visible precipitates in Figure 3 are associated with stable Mg_2_Ca precipitates [50]. In the case of ZN10 alloy (Figure 4), the Zn– and Nd–rich precipitates distributed within the entire microstructure (on the grain and twin boundaries and in the interior of the grains) are associated with the stable γ-phase Mg_3_(Nd, Zn) [50,51,52,53]. In the grain interior, pinning at the ordered GP zones (hcp, a = 0.556 nm, monolayer (0001)α disc) [50] takes place, as it has been identified using the TEM analysis in [54]. Moreover, a difference in segregation ability along TB in ZN10 is observed, marked by white and black arrows as segregated and non-segregated TB (STB and non-STB, respectively) in Figure 4a.

Despite differences in precipitation at TB and in the grain interior, immobile TB (i.e., those which persist at the same place before and after loading, marked by yellow arrows in Figure 3b and Figure 4b) can be observed in both alloys with further loading of the samples in the TMT state. However, some TB remain mobile (i.e., their position in the grain interior is changed as a result of the applied load, marked by green arrows) for both twin growth and shrinkage during re-compression and reverse tension. Moreover, mobile and immobile TB are commonly observed in the same grain. For compensating plastic deformation during re-compression, besides a continuous growth of existing twins, new twins can be nucleated (white arrows, Figure 3b). During reverse tension, detwinning is mainly realized by the movement of the existing TB (green arrows in Figure 3c and Figure 4c). However, similarly to that observed for Z1 alloy [49], during the reverse tension of both ZX10 and ZN10 alloys in TMT state, new twins are activated inside the existing twins with pinned TB (white arrows, Figure 3c and Figure 4c).

**Figure 5:**
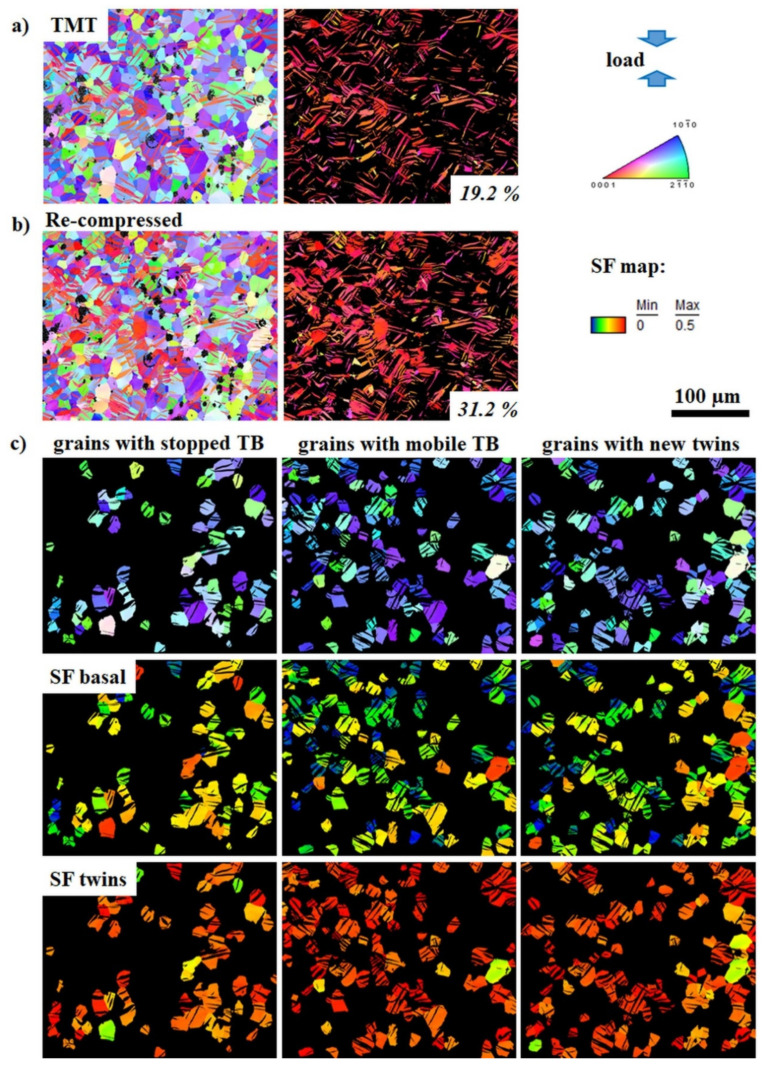
Orientation maps (entire microstructure and twins only) of the ZX10 alloy in the TMT state (**a**) and subsequent loading in compression up to 3% of strain (**b**). Grain grouping for TMT samples with respect to TB mobility (**c**) includes: first line–orientation maps, second and third line – Schmid Factor (SF) analysis for basal slip and extension twinning, respectively. Color code for orientation maps represents orientation with respect to ED.

**Figure 6:**
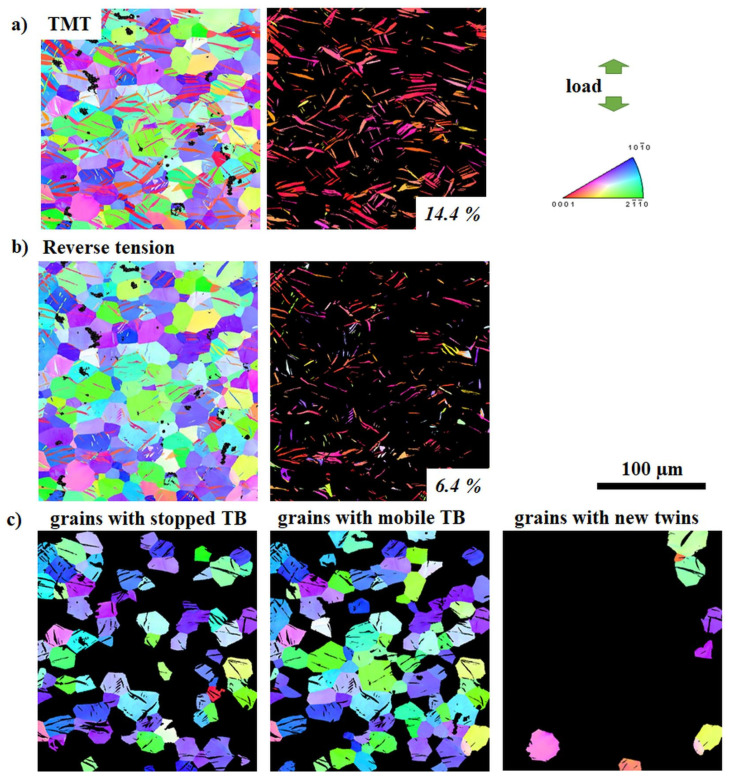
Orientation maps (entire microstructure and twins only) of the ZX10 alloy in the TMT state (**a**) and subsequent loading in tension up to 2% of strain (**b**). Orientation maps for grain grouping for reverse tensioned samples with respect to TB mobility (**c**). Color code for orientation maps represents orientation with respect to ED.

**Figure 7:**
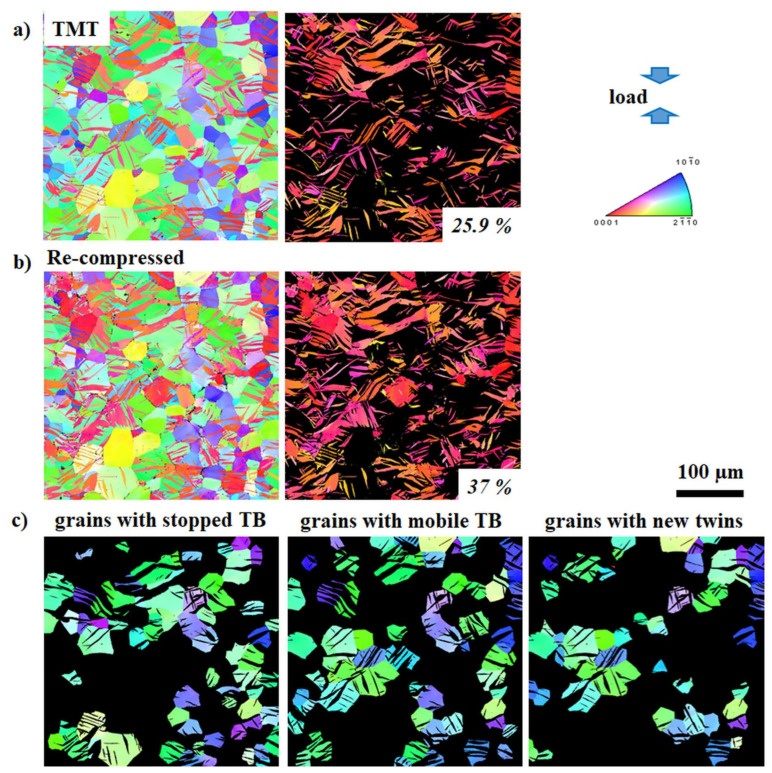
Orientation maps (entire microstructure and twins only) of the ZN10 alloy in the TMT state (**a**) and subsequent loading in compression up to 3% of strain (**b**). Orientation maps for grain grouping for TMT samples with respect to TB mobility (**c**). Color code for orientation maps represents orientation with respect to ED.

**Figure 8:**
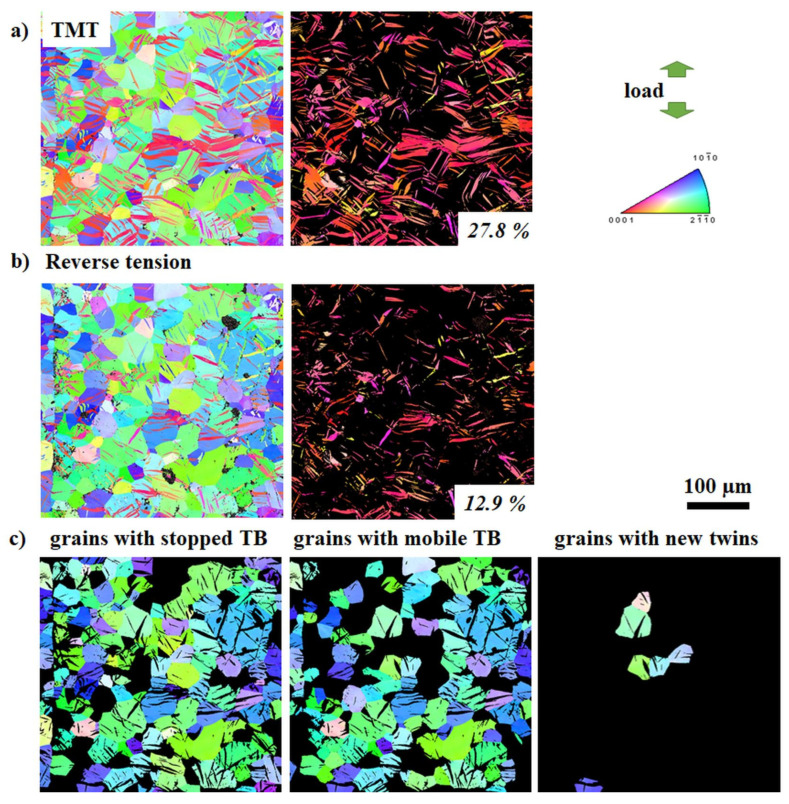
Orientation maps (entire microstructure and twins only) of the ZN10 alloy in the TMT state (**a**) and subsequent loading in tension up to 2% of strain (**b**). Orientation maps for grain grouping for reverse tensioned samples with respect to TB mobility (**c**). Color code for orientation maps represents orientation with respect to ED.

To evaluate the influence of twinning and detwinning impedance on the mechanical properties, the twin volume fraction (TVF) increment or decrement as a result of further loading of samples in TMT state can be used as a representative numerical parameter. It is based on the idea that twin growth or shrinkage (resulting in a certain increment or decrement of TVF) depends on the ability of TB to move. The nucleated extension {10-12} twins provide plastic compatibility for deformation along the *c*-axis according to the von-Mises criteria. Therefore, the achieved TVF is strongly related to the mechanical behavior of the material. The development of the TVF during re-compression or reverse tension of the ZX10 and ZN10 alloys after TMT are depicted in Figure 5, Figure 6, Figure 7 and Figure 8 (as a representative part of the microstructure). Maps containing only twins are presented separately for better visualization of the twin size and the TVF increment or decrement. The variation in the TVF values is included in the evaluation error. Moreover, the analysis of orientation maps provides information about the effect of parent grain orientation on further growth, shrinkage or “no change” of twins. Thus, the position of TB before and after loading was analyzed for each grain in the obtained orientation maps in order to reveal if those TB are either mobile or not with respect to the orientation of the parent grain.

The original TMT microstructure is a result of the pre-compression and heat treatment. Thus, a partly twinned microstructure with a TVF of 14–19% and 26–28% for the analyzed area are observed in the TMT samples of the ZX10 and ZN10 alloys, respectively, cv. Figure 5a and Figure 6a for ZX10 and Figure 7a and Figure 8a for ZN10. The applied pre-compression level provides a high number of twin boundaries enabling the analysis of their mobility in the scope of the present work. Twins in the ZX10 alloy appear thinner compared to those in the ZN10 alloy. It is not of special interest along with the considerations in this work that the isothermal aging procedure as a part of the TMT can affect the resulting TVF, e.g., by reducing its value as reported for ZX10 in [15]. All observed twins in the microstructure of ZX10 and ZN10 are consistent with {10-12} <10-11> extension twins with a reorientation of the original lattice by 86.3° (±5°). No further twin variants (e.g., compressive or double twins) having different misorientation angles were observed. Among the observed TB (all having misorientation of approximately 86°), there are pinned and non-pinned TB. Therefore, the effect of the misorientation angle of twinning on the pinning effect along TB is eliminated.

Following the initial TMT condition, the respective orientation maps after re-compression or reversed tension are shown in Figure 5b, Figure 6b, Figure 7b and Figure 8b, respectively. It becomes generally clear that re-compression leads to an increase of the TVF (ZX10: 31%, ZN10: 37%), but to a decrease after reversed tension (ZX10: 6%, ZN10: 13%). A detailed analysis of the maps reveals that during the following loading of the samples in the specified TMT state, there are few scenarios for the TB mobility. Therefore, the grains were separated into three groups: with stopped (at least one of TB is immobile) or mobile TB (including partly or completely vanished TB in case of reverse loading) and with newly activated twins. In both investigated alloys, there are immobile and mobile TB as well as newly activated twins. Orientation maps (first lines in Figure 5c, Figure 6c, Figure 7c, and Figure 8c) indicate that (im)mobile TB and the nucleation of new twins are rather independent on the orientation of the parent grains. Stopped TB are observed in both alloys after re-compression or reverse tension of sample in the TMT condition (grains with stopped TB in Figure 5c, Figure 6c, Figure 7c and Figure 8c). Therefore, as a result of intermediate isothermal aging, a number of TB remain immobile for both subsequent loading: re-compression or reverse tension. In general, the suppression of the TB mobility is independent on the loading direction. Besides immobile TB, some TB maintain their mobility for both forward and backward movement (grains with mobile TB in Figure 5c, Figure 6c, Figure 7c and Figure 8c). Moreover, the nucleation of new twins is observed to be independent on the loading mode in both the ZX10 and ZN10 alloys (grains with new twins in Figure 5c, Figure 6c, Figure 7c and Figure 8c). Naturally, there are newly activated twins during re-compression. However, during reverse tensile loading, despite the unfavorable lattice orientation for extension twinning (*c*-axis is perpendicular to tensile loading), some new twins are nucleated as well.

To obtain more information about the nature of twin behavior (nucleation of twins and TB mobility for its growth or shrinkage), the Schmid Factor (SF) values for the activation of basal slip and extension twinning have been estimated for each group of grains. For instance, the SF analysis for the TMT sample of ZX10 alloy subjected to re-compression is summarized in Figure 5c, lines labeled “SF basal” and “SF twin”. For all three grain groups, SF values for basal slip are comparable (in color scale in Figure 5c), as well as in the case of SF for twinning. Thus, the SF analysis does not reveal significant preferences for the activation of new twins or (im)mobility of existing TB in the analyzed groups. (SF analysis for reverse tensioned ZX10 and ZN10 samples, and re-compressed ZN10 samples are not presented in the manuscript.) No significant difference is observed with respect to the alloying content: SF values are comparable within different grain groups for both alloys, ZX10 and ZN10. The only general tendency that persists for all grain groups is the following: the SF values for twinning activity are higher for all sample states compared to those for a basal slip (see e.g., line “SF twin” vs. line “SF basal” in Figure 5c).

**Figure 9:**
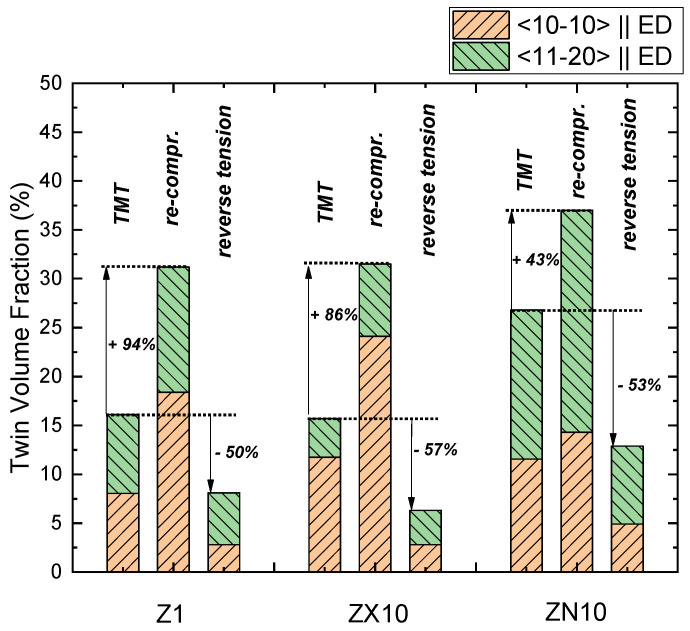
Twin volume fraction (TVF) development for two groups of grains with their <10-10> or <11-20> axis oriented parallel to the loading axis in the TMT state and subsequent loading of Z1 [49], ZX10 and ZN10 alloys. The evaluation error of TVF values for each sample is about 4%.

In order to explain the twinning–detwinning process in the TMT sample with respect to texture and grain size, the upcoming discussion also refers to the binary Z1 alloy without an addition of Ca and Nd, which has a texture comparable to the one of ZX10 but with significantly larger grain size. A detailed analysis of this alloy was reported previously in [49]. Thus, the TVF values for ZX10 and ZN10 alloys together with the data for a binary Z1 alloy [49] in different conditions are presented in Table 2, Figure 9. Taking into account a relatively high evaluation error (about 4%), for the samples in the TMT state, the ZN10 alloy is characterized by the largest TVF comparing to ZX10 and Z1 alloys. It is also noticeable that the TVF increments, as a result of re-compression variates, are the highest for Z1 and the smallest for the ZN10 alloy within the investigated alloys. The TVF decrement during reverse tensile loading is rather comparable for Z1, ZX10, and ZN10 alloys-about 50%, Figure 9.

Further, TVFs have been analyzed with respect to the orientations of the parent grain: its <10-10> or <11-20> axis is oriented parallel to ED, Table 2, Figure 9. The deviation of grain orientation from the specified direction has been set as 17°–20° in order to achieve the best separation of the grains into groups. This procedure has been performed by the same methodology as for the Z1 alloy reported in [49]. While in the Z1 alloy in the TMT condition, there is no significant difference in TVFs for those two groups of grains, the variation in TVF values for the ZX10 and ZN10 alloys is more pronounced: in ZX10, higher TVF is observed for <10-10> grains, while in ZN10, the tendency is rather opposite. Furthermore, besides the orientation effect of twin nucleation in this original TMT condition, the orientation effect is also observed for twinning–detwinning during re-compression or reverse tension after intermediate isothermal aging. In the case of ZX10, independently on the loading mode, higher twin activity (TB mobility and nucleation of new twins) is observed in grains having <10-10> axis parallel to ED: indicated by the larger TVF value in re-compressed and smaller TVF value in the reverse tensioned sample. The Z1 alloy exhibits a similar tendency: higher twinning activity in grains with a <10-10> axis oriented parallel to ED. While ZN10 shows a higher TVF value for grains having <11-20> axis parallel to ED in the TMT condition, during further loading (both re-compression and reverse tensile loading) the TB mobility, realized by propagating forward or backward in case of twin growth and shrinkage, is rather independent on the orientation of parent grain. The TVF increments and decrements for both groups of grains (<10-10>||ED and <11-20>||ED) are comparable in ZN10, Table 2 and Figure 9.

## 4. Discussion

The difference in CYS for the ZX10 and ZN10 alloys in the as-extruded state (Figure 2a, Table 1) can be explained by a variation in texture (intensity and distribution within poles) (Figure 1). Accordingly, the ZN10 alloy exhibits a stronger alignment of basal planes, i.e., the grains have rather preferable orientation for twinning during compressive loading along ED, which leads to a lower CYS. In the case of ZX10 with a weaker texture, i.e., less ordered alignment of basal planes, less significant extension twinning results in higher CYS. The difference in the texture is well reflected in the variation of TVF values being higher in ZN10 than in ZX10. For reference, the Z1 alloy, having a comparable texture to that in ZN10 (similar distribution of texture counterparts with slightly higher intensity at <11-20> pole leading the maximum intensity of 4.9 m.r.d. for ZN10), is characterized by significantly smaller TVF (16% vs. 25%) and lower CYS (56 vs. 77 MPa). It can be explained by the larger grain size in Z1 (~50 μm) compared to that in ZN10 and ZX10 alloys (12 ± 2 µm). Grain size is of key importance for twin nucleation: twins activate first in larger and then in smaller grains, i.e., twinning is grain-size dependent [34,35]. Moreover, there is a tendency that twins first propagate in length and then in thickness [26]. Therefore, by applying the same level of pre-strain, twins in smaller grains manage to go through the entire grain and continue to grow in thickness, while twins in larger grains propagate so far in length and have a small width. This suggestion is supported by the EBSD orientation maps indicating rather tiny thin twins in the Z1 alloy, which occupy a small area with respect to the rest (non-twinned) microstructure, Table 2 and Figure 9 and [49].

The variation in the distribution of texture intensities between the <10-10> and <11-20> poles is also reflected in the difference in the TVF for the respective groups of grains (their <10-10> or <11-20> axis is oriented parallel to ED, loading axis) for samples in the TMT state, Table 2. In ZX10, a higher TVF is estimated for <10-10>||ED grains, while ZN10 shows higher TVF in <11-20>||ED grains,-both alloys following “non-uniform” distribution in the texture between the <10-10> and <11-20> poles, Figure 1. Moreover, twins in ZX10 are thinner compared to those observed in ZN10. Preferential twinning activity in these two groups correlates to the SF estimation as follows. It was shown on pure Mg single crystals that compressive loading along <11-20> axis leads to a higher number of extension twins, realized by 4 variants with SF of 0.35, while during compressive stress along <10-10> axis, the activation of 2 variants with SF of 0.5 takes place [55]. Moreover, it was found that 4 variants with SF of 0.35 during compression along <11-20> axis lead to a steeper hardening slope. In the case of polycrystalline Mg alloys, the situation is more complicated, and aspects such as grain size, the existence of twins in neighboring grains, local distribution of strain, and other factors also contribute to the twinning activity. Nevertheless, the estimation of the SF for different twin variants is in agreement with experimentally observed data in the present work. Accordingly, in ZN10 with higher texture intensity at the <11-20> pole, twinning has a tendency to be realized by easily growing 4 twin variants (with high SF of 0.35 in the ideal case of single crystalline sample) and leads to a steeper hardening slope at the beginning of plastic deformation (up to 3% of strain, see Figure 2a) compared to a plateau for ZX10. In contrast, in ZX10 with more pronounced intensity at the <10-10> pole, twinning is realized by either easily activated two twin variants (SF of 0.5) or less favorable 4 variants with SF of 0.15. Nevertheless, the global texture needs to be taken into account, which is not that strong in ZX10 (compared to ZN10), and other deformation mechanisms (dislocation glide in various slip systems) are realized supporting higher CYS. The activity of other deformation mechanisms compensates and suppresses the twinning activity in ZX10, and the number of thin twins occupying a small area can be observed in the microstructure (Figure 5a and Figure 6a). Besides the variation in twinning activity, differences in the hardening slope (up to 3% of strain) also bring variation in the gained number of dislocations and, therefore, it has also an influence on the interaction of dislocations with the following isothermal aging.

Further, in Mg-Zn-(Ca/Nd) alloys containing various precipitates in the as-extruded state, the precipitation effect on strengthening should be considered. In general, in Mg alloys with twin-free microstructure (i.e., without pre-compression), precipitation in the matrix at GP zones or at the dislocations can suppress twin growth and families of thin twin lamellae are nucleated [40,46]. Thus, taking into account the low texture intensity and high SF (0.5) of the most likely activated twin variant, relatively high precipitation density in ZX10 alloy can also explain the formation of thinner twins (Figure 5a and Figure 6a) compared to those in ZN10. A more random texture and the presence of precipitates stimulate the activity of basal and non-basal dislocations in ZX10, resulting in high CYS. In contrast, in ZN10, plastic deformation is rather realized by twinning leading to low CYS. By applying higher stress, higher dislocation density can be achieved, generally higher precipitation is expected in ZX10 than in ZN10.

During further re-compression of the TMT samples, strengthening is observed in both ZX10 and ZN0 alloys, which finally leads to achieving a reversed YS asymmetry (CYS > TYS for TMT samples). For reference, softening in the Z1 alloy after application of the specified thermo-mechanical treatment leads to a compensation of the tension-compression asymmetry (TYS and CYS are equal) [49]. Among other facts, large grains in the Z1 alloy together with the thickness of nucleated twins in the alloy, i.e., twin microstructure refinement, have a significant impact on the yielding. In the microstructure of the Z1 alloy with large grains and thin twins, there is still enough free space for the realization of dislocation slip, twin growth and nucleation of new twins resulting in low YS. According to the study of a pre-strain effect on the TB mobility [12], low internal local strain in large grains with tiny twins gives rise for high TB mobility, while high local strains in small grains with multiple thick interconnected twins rather suppress the TB mobility. Therefore, the TB mobility in ZX10 and ZN10 in the TMT state is supposed to be lower than that for the Z1 alloy. Thus, for continuous twin growth in ZX10 and ZN10, higher stresses are required resulting in a significant strengthening effect. The effect of thermo-mechanical treatment on the YS and its tension-compression asymmetry in the extruded ZX10 alloy with different grain size has been reported in [9,15]. Nevertheless, the vanishing of the yield asymmetry between tension and compression can be achieved even without intermediate heat treatment, i.e., only by pre-compression itself [4]. Similarly, a reversed YS asymmetry was observed after sole pre-compression up to 4% in the case of pure Mg, AZ31, and AZ91 [11]. It should be also noted, that an increase in TYS and a decrease in CYS due to precipitation as a result of intermediate heat treatment naturally depends on the amount of solute elements, annealing time and temperature as well as the pre-compression level. For example, an increase in TYS by approximately 70 MPa can be achieved for AZ91 after 5000 s at 250 °C, while for Pure Mg and AZ31 an increase of TYS is about 10 MPa and 25 MPa, respectively [13]. Interestingly, that same increment in CYS of 25 MPa after applying different pre-compression level and followed by heat treatment has been observed in ZX10 [15] independently on the average grain size [9].

In general, during the re-compression of the extruded Mg alloys, the ongoing growth of twins requires the movement of TB into a grain area with a potentially high concentration of dislocations from the pre-compression. The dislocation density inside the twin is supposed to be low (because it was just created by the movement of the original TB). The dislocations in the non-twinned microstructure, as well as TB itself, act as good nucleation points for precipitates during heat treatment. Moreover, precipitation along TB is energetically more favorable than in the matrix, and precipitation-free zones along TB decorated by precipitates can be observed, e.g., in ZX10 alloy [15]. However, intermediate heat treatment brings not only precipitation but also relaxation of back stresses around the TB. Despite the hypothesis that the dislocation density inside the twin is lower than that in the parent grain, together with the relaxation effect caused by the heat treatment, the internal stress inside the twin and in the interior of the grain is comparable, and TB can remain to be mobile for both directions: twin growth and shrinkage (grains with mobile TB in Figure 3c and Figure 4c). At the same time, pinned TB with suppressed mobility for further movement are observed for both alloys. Thus, intermediate isothermal aging is able to segregate and completely stop TB. The restrained growth of existing twins and increased contribution of new twin nucleation as a result of annealing at 200 °C for 0.3–24 h has been shown, e.g., in Mg, AZ31, AZ91 [13], AZM110 [56]. A suppressed TB mobility has been determined by high back stresses, when a low annealing time is used for low alloying content samples, or by strong precipitation of solute atoms along TB (high friction stress) [13]. The strong influence of alloying elements and the applied aging on TB strengthening has been also revealed in AZ31, AZ61, and AZ91 alloys [57]. Strengthening by intermediate heat treatment of pre-twinned samples can be explained as follows: the stress required for the new twin nucleation and/or movement of pinned TB is larger than the stress for TB propagation in non-heat treated samples [11,42]. Thus, higher stress required for the movement of pinned TB and the activation of new twins contribute to the increase of the YS with respect to the pre-compression level, as can be seen in the case of both ZX10 and ZN10 alloys, Table 1.

In the case of the TMT samples, the twinning during re-compression is determined by the existing fraction of twins (TVF and local concentration of strain given by existing twins) and the ability of TB to move for twin growth with respect to the pinning effect. During re-compression of both alloys, ZX10 and ZN10, twinning is still the dominating mechanism, and multiple twinning is realized by the growth of existing twins and nucleation of new twins, Figure 5 and Figure 7. Slightly larger TVF increment during re-compression of ZX10 compared to ZN10 can be explained by a larger non-twinned area in the TMT state of ZX10. Despite differences in segregation and precipitation along TB (Figure 3a and Figure 4a), the same modes of TB mobility are observed in ZX10 and ZN10 as well as in a binary Z1 alloy [49]: mobile and immobile TB are observed during further loading of the TMT samples, Figure 3b,c and Figure 4b,c. Mobile TB remain their mobility for both, forward or backward movement independently on the alloying content and thus the types of precipitates. In contrast, pinned TB with suppressed mobility for further movement are observed in all alloys. It is highlighting that there are twins with mobile and immobile TB at the same time. It is supposed that the distribution of dislocations along TB and the resulting residual back stress concentration, as well as the ability for precipitation and relaxation during isothermal aging, determine the individual scenario of the TB mobility. The observed inhomogeneity in segregation along TB in ZN10 (segregated and non-segregated TB marked as STB-nonSTB in Figure 5a) is most probably caused by the geometry of the twins and the complexity of twin growth, particularly the distribution of TB dislocations, which could vary from one to another twin boundary [58,59,60,61,62]. Further detailed analysis in this regard is essential and will be presented elsewhere.

Nevertheless, the twinning activity, realized by the same TB mobility modes leading to a comparable increment in TVF for two alloys, does not explain the more pronounced strengthening in ZX10 than that in ZN10 (CYS increment is 23 vs. 9 MPa), Figure 2b,c. Thus, the dislocation density and precipitation in the TMT state have a different contribution to the hardening of the two alloys. First of all, a high dislocation density in ZX10 achieved by reaching higher stress during pre-compression up to 3% compared to that in ZN10 could lead to a strengthening effect. A higher dislocation density provides more nuclei for precipitation and, therefore, results in precipitation strengthening as well. Further information about the effect of the precipitate morphology (such as shape, lattice structure, size) on the strengthening and resulting mechanical performance can be found in [45,63].

During the reverse loading of the pre-compressed samples with twinned microstructure, the plastic deformation is controlled by detwinning [18,20,23,64]. The common case of detwinning in non-heat treated samples is a shrinkage of existing twins realized at the expense of the nucleation and propagation of new twins [20]. Cui et al. [11] indicated that detwinning is undertaken in a much easier way than twin growth due to the presence of the back stress after twinning. In the case of applied intermediate heat treatment, the situation differs from classical loading by the contribution of suppressed TB mobility. For example, Park et al. [41] have shown experimentally that detwinning at the early deformation is retarded by annealing. Nevertheless, delaying detwinning has been also observed in the non-heat treated samples [11,20]. Twin-twin junctions containing tilted prismatic-prism and basal-basal boundaries suppress or delay detwinning and lead to strain hardening even in non-heat-treated alloys [65].

Results of the present work indicate that independent of the alloy content, during reverse tensile loading of samples in the TMT state, new twins inside existing twins with pinned TB are formed, Figure 3c and Figure 4c. Most probably, the applied intermediate isothermal aging releases back stresses around such TB and for the compensating of the applied stress it is easier to nucleate new twins rather than move pinned TB. Nucleation of secondary twins inside existing ones has also been observed in [10]. The appearance of the identical twinning variant inside an existing twin has been predicted by modeling [7,30], and was observed experimentally in the binary Z1 alloy subjected to isothermal aging after pre-compression [49]. In addition, it was found that the nucleation of the new twin inside the existing one is more significant for Mg alloys compared to pure Mg [11].

However, this effect is not uniform, and during reverse loading mobile or immobile TB can be also observed in all investigated alloys, Figure 3c and Figure 4c. In Mg alloys with a Ca and Nd addition as well as in a binary Mg–Zn alloy, the migration of pinned TB can be realized either simultaneously from both TB or as a particular case – only one boundary is mobile. From a detailed analysis of BSE and EBSD data, it can be concluded that besides a global texture effect, the orientation of the parent grain (<10-10> or <11-20> is parallel to ED) or a SF does not provide a rule for preference in the (im)mobility of TB or the nucleation of new twin neither in the ZX10, ZN10, nor in Z1 alloys. Regarding the texture effect, during re-compression and/or reverse tensile loading, the TVF increment and decrement in two specified groups of grains resembles the tendency for the TVF in the TMT state. Particularly, a higher TVF increment in <11-20>||ED grains during re-compression or reverse tension of ZN10 is related to higher texture intensity at <11-20> pole. More pronounced TVF changes in <10-10>||ED grains during further loading of the Z1 [49] and ZX10 alloys (Figure 9, Table 2) are also caused by higher texture intensity at <10-10>-pole.

Thus, detwinning in pre-compressed samples with potentially pinned TB (due to intermediate isothermal aging) in Mg alloy with a Ca and Nd addition as well as in a binary Z1 alloy is realized by both routes: migration of pinned TB or nucleation of a new twin in the primary twin resulting in two or more separate residual twins [11,49]. The presence of different modes of detwinning is definitively the result of TB pinning.

Moreover, few newly activated twins inside the non-twinned interior of grains are observed despite generally unfavorably orientation for the extension twin activation during tension perpendicular to the *c*-axis. Those twins are identified as another variant compared to twins activated during pre-compression from six possible modes [2,20,66]. During pre-compression, the reorientation of the lattice caused by twinning leads to rotation of basal planes toward the compression axis, ED. While during tension, few twins can nucleate in the mode having basal planes rotated around the tensile axis, i.e., keeping basal planes basically parallel to ED. However, the newly created twins have a more favorable orientation (compared to a parent grain) for further dislocation slip, and consequently, extension twins can indeed compensate the tensile strain applied perpendicular to the *c*-axis. As there is no significant difference in the SF values, Figure 6c, the activation of such twins is individual and depends on a combination of local factors (such as internal stress concentration, mobility of existing TB in those grains, precipitation, etc.). Similar twin nucleation has been observed during reverse loading of pre-compressed non-heat treated AZ31 alloy, and the illustration of the twin variants can be found in [20].

Finally, despite the realization of detwinning by various modes, the same TVF decrement (about 50%) in ZX10 and ZN10 or binary Z1 alloys (i.e., independent on Ca and Nd alloying) is achieved independently on the grain size and the variation in the initial texture. The TVF decrement is controlled by a combination of stress levels of pre-compression and reverse tensile loading. The same decrement observed in all investigated alloys can be explained by a formation of the identical texture component produced by twins during pre-compression. Thus, this newly-formed texture component controls detwinning and results in similar TVF decrement and subsequently the same YS decrement for the investigated alloys. Nevertheless, dislocation and precipitation hardening during mechanical loading also contributes to the strengthening of the alloys. Similarly to re-compression loading, the contribution of dislocation and precipitation hardening is more pronounced for ZX10 comparing to ZN10. Therefore, the YS decrement during reverse loading is slightly smaller for ZX10 than that for ZN10 (23 vs. 31 MPa for ZX10 and ZN10 alloys, respectively).

## 5. Conclusions

The variation in the texture (alignment of basal planes parallel to ED and distribution of the texture intensity between the <10-10> or <11-20> poles) has a substantive effect on the twinning and twinning–detwinning processes, and, therefore, on the mechanical performance of the investigated alloys.

During re-compression and reverse tensile loading, despite differences in segregation and precipitation in the interior of grains and along twin boundaries (TB), the same modes of the TB mobility are observed in both ZX10 and ZN10 alloys as those indicated previously in a binary Z1 alloy without Ca and Nd alloying. The distribution of dislocations and resulting residual back stress concentrations along TB caused by pre-compression as well as the ability for precipitation and relaxation due to isothermal aging determine the individual scenario of pinned TB mobility. Thus, the segregation of solute atoms along TB resulted from a specified heat treatment does not completely suppress the TB mobility and they maintain the ability to propagate forward or backward. There are twins with mobile and immobile TB at the same time. During reverse loading of the investigated alloys, independently on the addition of 1 wt.% of Ca or Nd detwinning is realized by the migration of pinned TB and nucleation of new twins inside the existing twin lamella. During re-compression, continuous twinning naturally stimulates twin growth as well as the nucleation of new twins in the non-twinned interior of grains. Nevertheless, newly created twins in the interior of grains are also observed during reverse loading, which leads to a reorientation of the original lattice into a more favorable orientation for dislocation slip with keeping basal planes oriented parallel to ED, similar to the initial texture.

The increase and decrease of twin volume fraction were analyzed with respect to the texture and the TB mobility modes and correlated with yielding during further loading in compression or tension. A similar decrement in yield strength has been explained by the formation similar texture component (provided by twins), which controls the detwinning process in the ZX10 and ZN10 alloys as well as in a binary Z1 alloy.

Nevertheless, the deformation of the ZX10 and ZN10 alloys is determined not only by the contribution of the twinning–detwinning process, but also by the dislocation activity together with the precipitation effect caused by pre-straining and intermediate heat treatment. Particularly, despite the texture effect, more pronounced strengthening in ZX10 compared to ZN10 is associated with the differences in precipitation and dislocation density.

## Figures and Tables

**Table 1:** Values of yield strength (YS), σ0.2, for ZX10 and ZN10 alloys in as-extruded and in the TMT state. Symbols ↓ and ↑ represent a decrease or increase of CYS and TYS values with respect to pre-compression level (140 MPa for ZX10, 120 MPa for ZN10), i.e., softening or hardening, respectively. YS ± 1 MPa.

Yield Strength (YS), σ_0.2_, MPa		
Condition:	ZX10	ZN10
Initial	113	77
Re-compression of TMT	166 ↑	129 ↑
Reverse tension of TMT	117 ↓	89 ↓

**Table 2:** Twin volume fraction (TVF) for grains with their <10-10> or <11-20> axis parallel to the loading axis in the TMT state of ZX10, ZN10, and Z1 [49] alloys. The evaluation error of TVF values for each sample is about 4%.

Twin Volume Fraction, %		ZX10		ZN10		Z1
TMT (pre-compressed + heat treated) sample	Reference	Figure 5a	Figure 6a	Figure 7a	Figure 8a	[49]
	<10-10>||ED	12.1	11.4	9.9	13.2	8.05
	<11-20>||ED	4.8	3.1	16	12.6	8.05
	Total	16.9	14.5	25.9	25.8	16.1
Re-compressed of TMT	Reference	Figure 5b		Figure 7b		
	<10-10>||ED	24.1		14.3		18.4
	<11-20>||ED	7.4		22.7		12.8
	Total	31.5		37		31.2
Reverse tension of TMT	Reference		Figure 6b		Figure 8b	
	<10-10>||ED		2.8		4.9	2.8
	<11-20>||ED		3.4		8	5.3
	Total		6.2		12.9	8.1
